# Occurrence and Seasonal Variations of Aflatoxin M_1_ in Milk from Punjab, Pakistan

**DOI:** 10.3390/toxins11100574

**Published:** 2019-10-02

**Authors:** Naveed Akbar, Muhammad Nasir, Naureen Naeem, Mansur-ud-Din Ahmad, Sanaullah Iqbal, Anjum Rashid, Muhammad Imran, Tanweer Aslam Gondal, Muhammad Atif, Bahare Salehi, Javad Sharifi-Rad, Miquel Martorell, William C. Cho

**Affiliations:** 1 Department of Food Science & Human Nutrition, University of Veterinary and Animal Sciences, Lahore 54000, Pakistan; naveedagri@gmail.com (N.A.); nasir@uvas.edu.pk (M.N.); naureen.naeem@gmail.com (N.N.); sanaullah.iqbal@uvas.edu.pk (S.I.); 2 Department of Epidemiology & Public Health, University of Veterinary and Animal Sciences, Lahore 54000, Pakistan; mansuruddin@uvas.edu.pk; 3 Department of Dairy Technology, University of Veterinary and Animal Sciences, Lahore 54000, Pakistan; anjofst@gmail.com; 4 University Institute of Diet and Nutritional Sciences, Faculty of Allied Health Sciences, The University of Lahore, Lahore 54000, Pakistan; mic_1661@yahoo.com; 5 School of Exercise and Nutrition, Faculty of Health, Deakin University, Victoria 3125, Australia; tgondal@deakin.edu.au; 6 Department of Clinical Laboratory Sciences, College of Applied Medical Sciences, Jouf University, Sakaka 75471, Saudi Arabia; aatif003@gmail.com; 7 Student Research Committee, School of Medicine, Bam University of Medical Sciences, Bam 44340847, Iran; 8 Zabol Medicinal Plants Research Center, Zabol University of Medical Sciences, Zabol 61615–585, Iran; 9 Department of Nutrition and Dietetics, Faculty of Pharmacy, University of Concepción, Concepcion 4030000, Chile; 10 Department of Clinical Oncology, Queen Elizabeth Hospital, 30 Gascoigne Road, Kowloon, Hong Kong 999077, China

**Keywords:** Aflatoxins, AFM_1_, ELISA, raw milk, Pakistan

## Abstract

The manifestation of aflatoxins in feed and food is a major issue in the world as its presence leads to some health problems. This study investigates the incidence of aflatoxin M_1_ (AFM_1_) contamination in raw milk samples which were collected from Punjab, Pakistan. The Cluster Random Sampling technique was used to collect 960 milk samples from five different regions, and samples were collected every month. The AFM_1_ level in raw milk was analyzed by the ELISA technique. The findings demonstrate that 70% of samples exceeded the United States permissible maximum residue limits (MRL 0.50 µg/L), with an overall AFM_1_ level that ranged from 0.3 to 1.0 µg/L. AFM_1_ contamination varied with the season: The highest average contamination was detected in winter (0.875 µg/L), followed by autumn (0.751 µg/L), spring (0.654 µg/L), and summer (0.455 µg/L). The Eastern region exhibited the highest average AFM_1_ contamination (0.705 µg/L). Milk samples from the Northern region were found to be widely contaminated, as 86.9% samples exceeded the US MRL, followed by the Eastern region, with 72.3% samples being contaminated with >0.5 µg/L AFM_1_. The study indicated that the raw milk supply chain was heavily contaminated. Recommendations and remedial measures need to be developed by regulatory authorities to improve the raw milk quality.

## 1. Introduction

Aflatoxins belong to a widely studied group of mycotoxins. The major aflatoxins producing fungi are *Aspergillus flavus* and *A. parasiticus* [1]. Aflatoxin contamination in food, feed, and agricultural produce is a matter of colossal concern around the world because of their carcinogenic, metabolic, mutagenic, immunosuppressive, and teratogenic effects [2,3]. Four major types of aflatoxins (B_1_, B_2_, G_1_, and G_2_) can contaminate food and feed, posing serious health complications for human beings, as well as for animals [4]. Aflatoxin M1 (AFM_1_) is the monohydroxylated derivative of aflatoxin B_1_ (AFB_1_), developed in the liver of lactating animals during metabolism and further excreted into the raw milk of cattle usually fed with AFB_1_-contaminated feed [5]. The International Agency for Research on Cancer (IARC) classified AFM_1_ as a 2B carcinogenic group because it can damage DNA and may cause various types of cancers [6]. Chromosomal anomalies, cell transformation in mammals, and gene mutation are also a few of the known outcomes of AFM_1_ exposure [7].

Milk is one of the nutritious sources required for the better growth of infants and children, and at the same time, it is a rich source of nutrition for all age groups [8]. However, with an increasing demand for milk, it becomes challenging for the dairy sector to maintain a uniform and standardized quality in under developed countries. This situation is posing serious health threats to consumers. Research studies have verified that pasteurization, heat processing, and a few other techniques are ineffective for controlling AFM_1_ in raw milk [9,10]. Once aflatoxins contaminate the milk supply chain, it becomes impossible to completely purify raw milk [11,12]. The consumption of AFM_1_-contaminated dairy products would likely harm human health [10,13]. Recent studies have highlighted the alarming threats to health associated with the use of milk contaminated with aflatoxins. This is the reason why the detection of aflatoxins in agricultural commodities and development of effective strategies for their control are key research areas in the world [14]. The lethal nature and harmful impacts of aflatoxins on the health of humans and animals have increased the need for effective management. Furthermore, it is also evident that the occurrence of aflatoxins in the food supply chain is also affected by the season, weather, and contaminated feed ingredients [11,15,16].

Many countries have set a maximum residue level (MRL) in milk to ensure food safety. The MRL of AFM_1_ varies worldwide as agricultural practices, milk collection systems, and the food supply chain are different in various parts or regions of the world. The European Union (EU) established a principle of “As Low As Reasonably Achievable” (ALARA) regarding aflatoxin levels in food products. The ALARA implies that the maximum permissible level of AFM_1_ in milk in the EU is 0.05 µg/L [17], which is one tenth of the restrictions implemented in the United States (US) [18], Brazil [19], and China [20], and is also lower than Syria, where the limit is confined to 0.2 µg/L and 0.05 µg/kg for raw milk and powdered milk, respectively [21]. According to the report published in 2002, about 100 countries have set limits of AFM_1_ [2], while there are still many countries in the world which do not have regulations for AFM_1_ in raw milk and milk-based products [22].

Pakistan is the third largest milk producer in the world, with 40 billion liters of milk production annually [23]. During 2016, about 43,818 metric tons of milk was consumed nationwide [24], while the milk supply and demand gap is still wide. Despite the immense potential, a sizeable bulk is untapped in Pakistan due to ineffective and informal milk marketing. The major chunk of milk demand (94%) is being fulfilled through the casual supply chain (milk-man), while the packed milk industry bridges only 6%. This informal supply chain is likely to bear a high contamination of AFM_1_. As several studies have reported, AFB_1_-contaminated feed is the source of milk AFM_1_ contamination [25]. The consumption of these contaminated feed sources is more common in an informal supply chain compared to corporate farming [26]. Recently, Pakistan imposed 5 µg/L legal limits of the AFM_1_ level in milk for processing and 0.5 µg/L in milk for consumers, but the implementation is faulty due to the lack of strict monitoring facilities. This study was aimed at investigating the occurrence of AFM_1_ levels in widespread milk supply chains of Punjab regions by examining the milk throughout the year. Several efforts have been made to investigate the AFM_1_ contamination in milk of Punjab, Pakistan, but all of these studies were limited due to the sample size [27], area [28], season [15], milking time [29], and point of sample collection [28]. However, this study was conducted with the screening of the raw milk supply chain in a wider area, which constituted the major portion of milk supply network, and included all seasons of Punjab, Pakistan. 

## 2. Results 

**Figure 1:**
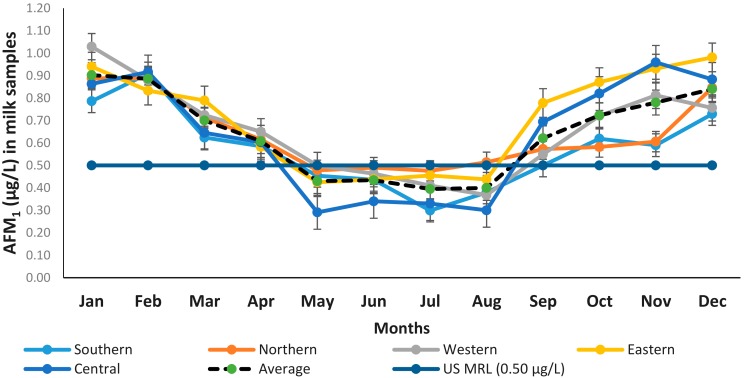
Occurrence of aflatoxin M_1_ (AFM_1_) (µg/L) in raw milk among five regions of Punjab, Pakistan, during the year 2015 (n = 960).

**Figure 2:**
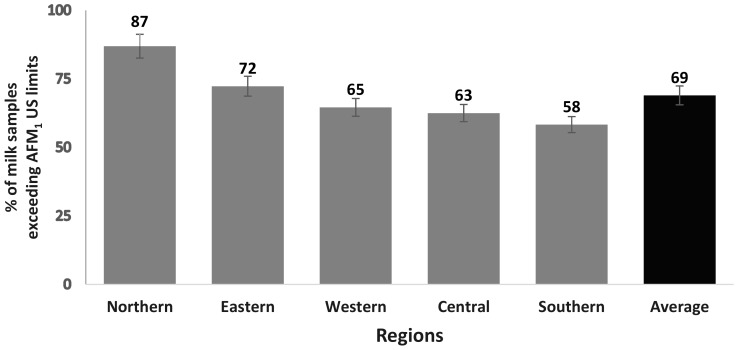
Percentage of milk samples exceeding the US AFM_1_ maximum residues level (<0.5 µg/L) throughout the year, in five regions of Punjab, Pakistan.

The occurrence of AFM_1_ in raw milk samples (n = 960) collected from five regions of Punjab is presented in Figure 1, and the total number of samples per month in the regions exceeding the US maximum residual limit are shown in Table 1. The proportion of AFM_1_-contaminated milk samples was higher than 50% in all regions (Figure 2). The average value of AFM_1_ (0.705 ± 0.211) in milk samples was found to be higher in the Eastern region, which was significantly (P < 0.05) different from the average value of AFM_1_ in the Southern region (0.577 ± 0.219) and on par with the average of AFM_1_ in the Western (0.655 ± 0.193), Northern (0.639 ± 0.150), and Central (0.577 ± 0.219) regions. The value of AFM_1_ was found to be statistically non-significant for the Western, Northern, and Central regions.

In the current study, the findings were segregated in terms of regions, and the highest concentration of AFM_1_ was observed in the Eastern region, while the lowest was found in the Southern region (Table 1). The results of the Eastern region showed that the maximum range (0.52–1.67 μg/L) of AFM_1_ contamination in milk samples occurred during the month of November, and the lowest (0.16–0.63 μg/L) was seen in the month of May. Similarly, the maximum range of AFM_1_ contamination in other regions, i.e., the Western (0.13–1.56 μg/L), Northern (0.54–1.5 μg/L), and Central (0.36–1.63 μg/L) regions, occurred in the months of Nov–Dec. The contents of AFM_1_ in milk samples varied from 0.30 to 1.0 μg/L (Figure 1). The milk samples’ AFM_1_ contamination in all regions swiftly decreased from March to August. It was observed that AFM_1_ trends in all regions were almost similar to each other; however, after mid-April to August, the concentration remained below the US MRL (<0.5 μg/L). Immediately after August, a drastic increase in the AFM_1_ concentration in all regional milk samples was observed. The maximum contaminated samples (93.7%–100%) were in the month of February and the lowest (12.5%–43.7%) were in July.

One-way analysis of variance (ANOVA) was applied; to check for significant differences among the means of clusters and seasons, the Tukey HSD test was used. The results were considered statistically significant at p < 0.05. The clusters with the same superscript letters are statistically non-significant. 

This study showed that, on average, 69% of milk samples displayed AFM_1_ contamination that was more than 0.5 μg/L US MRL. The highest contaminated samples (87%) were in the Northern region and the lowest (58%) were in the Southern region (Figure 2).

The extent of detection of AFM_1_ was at the maximum during winter (0.875 µg/L), followed by autumn (0.751 µg/L), spring (0.654 µg/L), and summer (0.455 µg/L). The results given in Table 2 showed that irrespective of regions, 54%–88% of milk samples were contaminated with AFM_1_ in the range of 0.5–1.00 µg/L for the winter, autumn, and spring season. Additionally, in the summer season, 54%–78% milk samples in all regions were below the US MRL, i.e., <0.5 µg/L. 

## 3. Discussion

AFM_1_ contamination of food and feed is a global issue, especially in developing countries. The Punjab province of Pakistan is the major cash crop-producing and livestock-keeping area [30]. AFM_1_ contamination screening in the milk of this area depicts that the average AFM_1_ contamination in all regions of Punjab was above the recommended US regulations (0.5 μg/L). These findings are similar to those of Jawaid, et al. [31], who determined the AFM_1_ contamination in milk from the Sind province of Pakistan using ELISA and found 94% contaminated samples, such that 70% of samples surpassed the established standards of US regulations. Asghar, et al. [32] collected 156 fresh milk samples from local markets of Karachi, Pakistan, during 2016–2017. The results of these samples were analyzed by the ELISA technique and the mean contamination of AFM_1_ in milk was reported as 0.34 µg/L, with 32.7% samples being above the US allowable limits. Hussain, Anwar, Asi, Munawar and Kashif [27] performed HPLC with a fluorescent detection test to probe AFM_1_ contamination of the milk of cow, buffalo, camel, goat, and sheep. The contamination ranged from 16.7% to 34.5% for the five species. The severity of AFM_1_ contamination in Pakistani milk was further reported by Iqbal, Asi and Jinap [15], who analyzed 221 samples by HPLC and reported 45% contamination during the winter season and 32% contamination during the summer. Another study conducted in Central areas of Punjab Pakistan, found that 53% and 43% of buffalo and cow milk samples were contaminated, with an average AFM_1_ of 0.03 and 0.04 μg/L, respectively [22]. The findings further inferred that the AFM_1_ concentration was higher in milk samples obtained from urban or semi-urban areas compared to those from rural localities [22].

Many contemporary studies conducted across the world, especially in Asia, agree with the findings of the current study. Studies from border-sharing neighbor countries like India, having a similar geography, have reported ample incidences of AFM_1_ contamination in milk samples. Rastogi, et al. [33] carried out a competitive ELISA technique to analyze 87 milk samples and infant milks collected from the Indian market and found that 99% of samples were contaminated with AFM_1_ levels higher than the EU standard limits, whereas 9% of samples surpassed the US regulatory levels. The results of the current study are supportive of the reviews of Nile, et al. [34], where 46% of samples from different milking animals were contaminated. The mean value of AFM_1_ in buffalo, cow, goat, and sheep milk was 0.026, 0.018, 0.014, and 0.017 μg/L, respectively. In comparison to Pakistan, India has lower milk contamination issues; however, the difference in weather conditions, geography, and socio-economic status of both countries is not huge.

The climatic changes in Pakistan are the major reasons for AFM_1_ presence in the food supply chain. Usually, under climatic variations, farmers and farm owners face feed shortage and use alternatives, which become a source of AFM_1_ contamination. Cotton is the main crop of the Southern region, whereas corn is that of the Northern region. Corn dried under the sun in the open fields of Punjab, Pakistan, and stored in conditions that are favorable for fungal growth, results in the higher occurrence of AFB_1_. When the AFB_1_-contaminated sources are fed to the animals, they are secreted in the form of AFM_1_ in milk. In general, the weather conditions of the Southern and Northern regions are distinctly different due to variation in altitude. Hence, the Southern region is at the height of 400 ft, with a hot and dry climate, whereas the Northern region is around 2000 ft above the sea level and generally ambient and moist. The level of AFM_1_ was highly correlated with the topography [35] of the country, as indicated by the findings that contamination (%) was the highest in the Northern region and kept on decreasing, moving through to the Southern region (Figure 2). 

The results were further compared with studies from another border-sharing countries, i.e., China. Raw milk and other dairy products (135 samples) from Northeast China revealed that 41% of samples were contaminated, with an average AFM_1_ concentration ranging from 0.32 to 0.50 μg/L [36]. In other research, an attempt was made to examine the 72 raw milk samples from China, which reported that 59.7% of samples were contaminated, with an AFM_1_ concentration ranging from 0.01 to 0.42 μg/L [16]. Further findings identified that the concentration of AFM_1_ was higher during the winter season (October–February). It is significant to compare the findings from the present study to those of China as both nations are neighbors and share more or less the same weather conditions. The results of the present study were further compared with Iran, which shares a border and weather conditions as well. Kamkar [37] found the presence of AFM_1_ in 76.5% of raw milk samples, with concentrations between 0.02 and 0.28 μg/L. The study reported that 40% of samples exceeded the EU maximum tolerance limit. An increased concentration of AFM_1_ was found during winter and it was shown to be inversely proportional to the temperature [15]. In another study performed by Hashemi [38], it was shown that the temperature had no role in the contamination of AFM_1_, but the study by Kamkar [37] reported a positive correlation between these two variables. Our results are in agreement with those of Kamkar [37]. Another study conducted in Pakistan found a higher concentration of AFM_1_ in the winter season [39] compared to the other seasons and these findings are in agreement with the present and previous studies [37]. It is worth mentioning that no study related to milk contamination with AFM_1_ was found in Afghanistan.

Numerous contemporary research studies across the globe have reported the contamination of AFM_1_ in milk. There are many reports on AFM_1_ contamination in milk, including results from Slovenia [40], North Africa [41], Turkey [42,43], Brazil [44], Portugal [6,45], Morocco [46], Syria [21], Sudan [47], Serbia [48], Spain [49], Korea [50], and Croatia [51]. 

In the current study, AFM_1_ contamination in all regions portrayed a similar trend, which indicated that it was being influenced by common contributors. It was clear from Table 2 that the amount of AFM_1_ contamination in milk was significantly higher in the winter season (0.875 ± 0.406 µg/L) compared to the autumn (0.751 ± 0.148 µg/L), spring (0.654 ± 0.037 µg/L), and summer (0.455 ± 0.052 µg/L) seasons. A lower amount of AFM_1_ contamination was found in the summer season, which was significantly different from all other seasons. The current study revealed that the type of animal feed and its handling are two major contributors to raw milk contamination. For example, the handling of cotton seed cake and corn, which are traditionally and nutritionally essential feed ingredients and valued throughout the year. The harvesting, drying, and storage of these feed ingredients in humid weather increases the concentration of toxins in feed and ultimately become the cause of higher AFM_1_ in milk [11,16,52]. The current findings are in line with the study of Hussain, Anwar, Munawar and Asi [22], which demonstrated that an inadequate availability of green fodder and excessive usage of concentrated feed and some of its ingredients, such as corn, wheat straw, cottonseed cake, paddy straw, and wheat bran, increase the AFM_1_ contamination in milk.

Previous studies [39,53] have pointed out that the contents of AFM_1_ are at the maximum during winter and autumn compared to other seasons. Higher AFM_1_ contamination in milk during winter is similar to the milk samples studied in Iran during winter (average 0.121 μg/L) [54]. Ghajarbeygi, et al. [55] reported similar findings of higher contamination during winter in Iran to those presented in Croatia [51,52]. In another study conducted in Pakistan by Asi, Iqbal, Ariño and Hussain [39], higher levels of AFM_1_ were documented in the winter season compared to the summer season, with significant difference (*p* < 0.05). The present study results are in exact agreement with previous studies conducted in Pakistan [15,22,27,31,35,56], where they reported higher contamination of milk with AFM_1_ in the winter season. The present study found high levels of AFM_1_ contamination in milk samples collected in the autumn season (0.751 μg/L) after winter (0.875 μg/L); hence, the results are in agreement with those of Aslam, et al. [57], who reported significantly higher AFM_1_ levels in milk during the autumn season (2.60 μg/L). Similar studies conducted by Hojjatollah and Sepideh [58] also reported an increased level of AFM_1_ contamination in milk during the winter and autumn seasons, with an average value of 54.24 and 34.92 ng/L, respectively. Winter and autumn are both critical seasons indicating the maximum presence of AFM_1_ (Table 2). At the same time, the majority of milk samples had higher levels of AFM_1_ contamination (than US permissible limits), regardless of seasons and regions (Table 1 and Table 2). 

In a recent study, Miocinovic, et al. [59] concluded that AFM_1_ contamination in milk is greater in the autumn season. The results infer that the contamination of AFM_1_ in milk is influenced by seasonal variations, as reported in previous studies [39,60]. Jalili and Scotter [61] assessed the association between AFM_1_ contamination and feed consumed by animals in different seasons. The feeding pattern of animals more often alters from season to season. For instance, a number of research studies [22,62] have pointed out that the contamination of milk with AFM_1_ was influenced with a seasonal impact. The level of contamination in milk was observed to be greater in the cold season compared to the hot season in some other places [22,37,63]. 

Owing to the insufficient availability of feed during winter, farmers have to supplement animal feed, which is composed of various feed ingredients. The compound feed as a practice is prepared with discarded sources of grains and hence is susceptible to increases in aflatoxin levels in storage. In Pakistan, Asi, Iqbal, Ariño and Hussain [39] reported a higher AFM_1_ concentration in the milk of animals usually fed with compound feed compared to lactating animals fed on grazing or fresh green feed. It is a reality that animals have fractional access to grassland or pastures. Diet requirements are met with feed supplements. In addition to these factors, a higher milk yield in winter is a motivator for farmers to feed maximum inputs to animals that are in the form of corn, cotton seeds or cotton seed cake, raw rice bran/rice polish, wheat bran, and gluten. These ingredients are most vulnerable to fungal attack [22,36] and give the maximum output in terms of milk yield in winter. Another factor linked to the higher contamination in winter is the crop harvesting time. In Punjab, Pakistan, the corn-harvesting time is October, while the cotton-harvesting time is August to September, so they become a cheaper source of feed in these months. Dairy farmers grow these crops and feed animals without knowing their aflatoxin contamination. The commercial dairy farmers and farm farmers do not have the concept of standardized and hygienic feed usage. However, there are quite of few research studies which have shown no difference in AFM_1_ levels in samples obtained during winter and summer seasons [15,39,55,58]. At the same time, the results of these studies confirmed that the contamination of milk with AFM_1_ might be influenced by seasonal variations in different geographical locations. Despite the above factors, the weather conditions in Pakistan are unpredictable and variations in weather throughout the year may bring about abrupt changes in the contamination of AFM_1_.

## 4. Conclusions

The strength of this study lies with the results being obtained from a considerable number of samples (n = 960), which were collected during four seasons. In the current investigation, raw milk samples were collected from January to December. This research-based investigation confirms that the level of AFM_1_ in raw milk in Pakistan is due to erratic weather conditions and elevated levels of AFB_1_ contamination in animal feed. Regarding the seasonal impact on AFM_1_ in all regions, high levels were observed in the winter season, particularly from November to February. These observations are in agreement with previously published studies of elevated levels of AFM_1_ in winter. The increase in AFM_1_ level was higher compared to previous studies conducted in Pakistan, which shows a mounting concern of AFM_1_ in Pakistan. This study urges food authorities and livestock departments of Pakistan to mobilize food safety laws in the milk supply chain and initiate official regulations for the control of AFB1 in animal feed sources. Government agencies should introduce subsidy programs to introduce good storage practices, pre- and post-harvest control measures, effective grain drying techniques, and support to protect animal feed ingredients from the weather. The introduction of contamination-free forage sources, e.g., high-yield green fodder varieties, grasses, and silage-making techniques, could restrict AFM_1_ in the milk supply chain. Linkages between grain producers, feed manufacturers, dairy farmers, food, and livestock departments represent a dire need to combat increasing levels of contamination.

## 5. Materials and Methods 

### 5.1. Sampling Plan

**Figure 3:**
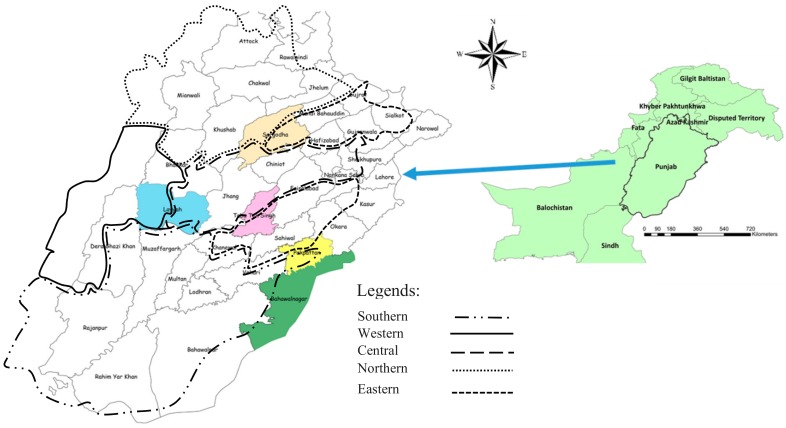
Geographical locations of milk sampling sites in different colors from five selected regions of Punjab, Pakistan. Data Source ArcGIS Map 10.3.

The Punjab province consists of thirty-six districts, which were divided into five regions, as follows: North (Attock, Chakwal, Rawalpindi, Sargodha, Khushab, Mianwali, Mandi Bhaudin, Gujrat, and Jhelum), South (Bahwalpur, Bhawalnagar, Multan, Khanewal, Lodhran, Muzaffargarh, Rahimyar Khan, Rajanpur, and Vehari), East (Gujranwala, Hafizabad, Pakpattan, Okara, Sahiwal, Kasur, Sailkot, and Norrowal), West (Dera Ghazi Khan, Bhakkar, and Layyah), and Central (Lahore, Faisalabad, Jhang, Toba Tek Singh, Chiniot, Sheikhupura, and Nankana Sahib), as shown in Figure 3 [64]. Sampling selection consisted of three stages. In the first stage, a district was selected from each region using Cluster Random Sampling. At the second stage, only one tehsil (an administrative area) was selected from each district. In the third stage, a milk shop was selected from each tehsil using random sampling. Milk samples were procured from local milk shops every month for one year.

### 5.2. Sampling

In this study, during the year 2015, a total of 960 milk samples were collected from 16 shops at random from each of the clusters (North, South, East, West, and Central) of Punjab, Pakistan. The plastic bottles, with a volume capacity of 200 mL, were filled with milk samples and placed in sampling coolers with icepacks to ensure that the temperature of the milk samples stayed below 10 °C. In most of the cases, samples were analyzed immediately; however, where the delay was unavoidable, samples were stored in a refrigerator.

### 5.3. Aflatoxin Determination 

The competitive enzyme immunoassay using ELISA kit Ridascreen^®^ Fast Aflatoxin M_1_, R5812 (R-Biopharm AG, Darmstadt Germany) was used for the quantitative analysis of AFM_1_ in the milk samples. Sample preparation was done by following the instructions given in the manual. Fifty (50 μL) samples or standards were added to a 96-well plate. Conjugate and anti-aflatoxin antibody solutions were added (50 μL) to each well. The solutions were mixed gently without splashing reagents from the wells by back and forth sliding on a flat surface for 10–20 seconds and incubated for 10 minutes at room temperature (20–25 °C). Liquid contents of the antibody wells were poured out by tapping the microwell holder upside down vigorously (three times in a row) against absorbent paper to ensure the complete removal of liquid from the wells. Again, each well was filled with 250 μL of washing buffer using an 8-channel pipette and poured out, and this step was repeated two times. After washing, 100 μL of substrate was added to the wells and gently mixed, as done previously, and incubated at room temperature in the dark for 5 minutes. Finally, the reaction was stopped by adding 100 μL of stop solution to each well and mixed gently, avoiding the splashing of reagents. Absorbance at 450 nm was measured by an ELISA plate reader (ELX800, Bio-Tek Instruments, USA).

### 5.4. Method Validation

The method was validated according to the criteria set by Commission Regulations 401/2006 [65]. The validation parameters were calculated in terms of linearity, limit of detection (LOD), limit of quantification (LOQ), linearity, repeatability (precision), and intermediate reproducibility (intra laboratory reproducibility), expressed as the relative standard deviation (RSD%) and trueness as recovery (Table 3 and Table 4).

Linearity was tested based on the six-point calibration curve acquired for an intermediate reproducibility (iR basis). Calibration curves were constructed as follows: the logit value for each standard was plotted against the decadic logarithm (log10) of the corresponding concentration. The logit value for each standard (log it STDX) was calculated using the following equation:

(1) $$\log it_{STDX}=\ln\frac{OD_{STDX}}{OD_{STD0}-OD_{STDX}}$$

where OD_STDX_ is the average optical density (duplicate measurements) of the standards recorded at 450 nm, and OD_STD 0_ is the average optical density (duplicate measurements) of the standard containing no AFM_1_ (0.00 µg/L) recorded at 450 nm.

**Figure 4:**
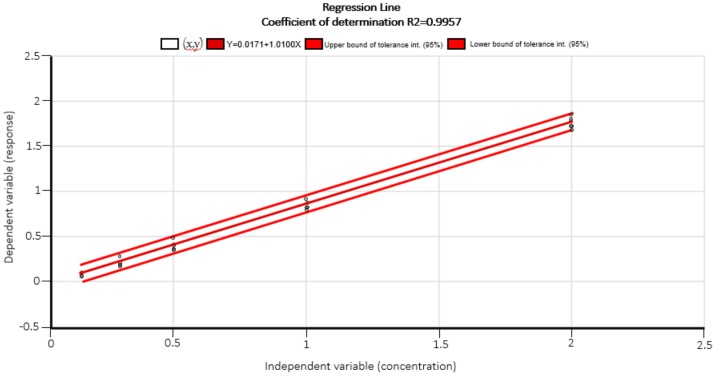
The plot of the regression line shows a linear response (r^2^ > 0.99) within the working range studied (0.125 µg/L to 2.00 µg/L).

A standard curve was constructed each time using series of standards of AFM_1_ solutions provided with the test kit. The sample concentration was calculated based on the standard curve (Figure 4). The plot of the regression line showed a linear response (r^2^ > 0.99) within the working range studied (0.125 to 2.00 µg/L).

In order to calculate the precision, milk was analyzed without any purification step and after spiking at three different AFM_1_ concentration levels (0.00, 0.25, 0.50, and 0.75 µg/L), on six different days, and each time in duplicate. Consequently, the number of AFM_1_ determinations at each level was 12, which gave 48 ELISA measurements for this matrix. All repeatability and intermediate reproducibility values (i.e., CV(r) and CV (iR), in %) were within 7.0% to 9.8% and 5.4% to 24.6%, respectively. Recoveries were calculated using the results obtained from the repeatability and intermediate reproducibility. All recovery values were within 79.3% to 86.0%, with relative standard deviation (RSD%) from 9.6% to 13.1% and the limit of detection (LOD) of 0.10 µg/L.

### 5.5. Statistical Analysis

Numerical variable data were presented as frequencies, percentages, and the average ± standard deviation. The bar chart was used to express the total % of AFM_1_ samples exceeding US MRL <0.5 µg/L in selected clusters, while the line chart indicated levels of averages of AFM_1_ (µg/L) in different months of the year 2015. One-way analysis of variance (ANOVA) was applied; to check for significant differences among means of clusters and seasons, the Tukey HSD test was used. The results were considered statistically significant at *p* < 0.05. Correlation and regression analyses were applied to calculate R^2^ using the statistical package Q Stat.net.

## Figures and Tables

**Table 1:** Aflatoxin M_1_ range (µg/L) and percentage of milk samples exceeding the US maximum residues level (MRL <0.5 µg/L) of aflatoxin M_1_ in five clusters of Punjab, Pakistan, during 2015 (n = 960).

Cluster	Total Samples	Months													Average AFM1 ± SD
			January	February	March	April	May	June	July	August	September	October	November	December	(µg/L)
Eastern	N = 192	% samples exceeding US MRL	93.7	93.7	75	68.7	56.2	25	43.7	43.7	75	100	100	93.7	0.705 ± 0.211^a^
		AFM1 range (µg/L)	0.35–1.64	0.45–1.65	0.34–1.35	0.17–0.87	0.16–0.63	0.18–0.78	0.24–0.67	0.15–0.65	0.25–1.63	0.54–1.56	0.52–1.67	0.46–1.65	
Western	N = 192	% samples exceeding US MRL	100	100	68.7	75	43.7	37.5	31.2	12.5	62.5	81.2	87.5	75	0.655 ± 0.193^a,b^
		AFM1 range (µg/L)	0.55–1.46	0.68–1.06	0.33–1.23	0.32–0.92	0.23–0.92	0.29–0.74	0.22–0.66	0.19–0.56	0.19–0.99	0.17–1.44	0.13–1.56	0.32–1.46	
Northern	N = 192	% samples exceeding US MRL	100	93.7	87.5	87.5	62.3	68.7	68.7	68.7	81.2	87.5	75	100	0.639 ± 0.150^a,b^
		AFM1 range (µg/L)	0.5–1.35	0.46–1.56	0.45–1.33	0.45–0.95	0.19–0.75	0.21–0.78	0.24–0.76	0.15–0.94	0.24–1.19	0.43–0.75	0.22–0.96	0.54–1.50	
Central	N = 192	% samples exceeding US MRL	100	100	87.5	62.5	0	12.5	12.5	6.2	81.2	100	100	87.5	0.637 ± 0.249^a,b^
		AFM1 range (µg/L)	0.55–1.44	0.56–1.43	0.46–0.85	0.35–1.23	0.16–0.47	0.12–0.64	0.16–0.66	0.04–0.89	0.34–1.59	0.64–1.00	0.65–1.56	0.36–1.63	
Southern	N = 192	% samples exceeding US MRL	87.5	100	75	81.2	31.2	18.7	0	18.7	56.2	75	75	81.3	0.577 ± 0.219^b^
		AFM1 range (µg/L)	0.47–1.14	0.56–1.3	0.43–0.88	0.33–0.99	0.23–0.75	0.3–0.65	0.19–0.49	0.13–1.13	0.19–0.84	0.26–1.3	0.13–0.87	0.40–1.50	

Region wise, 16 samples were analyzed monthly in the year 2015. In total, 192 samples/region/year were analyzed. The total number of samples was 960. Underlined values are the ranges of the lowest and highest AFM1 (µg/L).

**Table 2:** Incidence of AFM_1_ in raw milk samples from five regions of Punjab, Pakistan, with respect to season.

			Range of AFM_1_ Concentration (µg/L)	Southern	Northern	Western	Eastern	Central	Average AFM_1_ + SD
Winter	Average AFM_1_ (µg/L)			0.808 ± 0.273	0.874 ± 0.273	0.888 ± 0.284	0.918 ± 0.344	0.888 ± 0.275	0.875 ± 0.406 ^a^
	n = 240	Numbers of samples (% frequency distribution)	<0.50	5(10%)	2(4%)	4(8%)	3(6%)	2(4%)	
			0.51–1.00	30(63%)	32(67%)	27(56%)	26(54%)	34(71%)	
			>1.00	13(27%)	14(29%)	17(35%)	19(40%)	12(25%)	
Autumn	Average AFM_1_ (µg/L)			0.605 ± 0.206	0.594 ± 0.155	0.767 ± 0.344	0.902 ± 0.332	0.890 ± 0.188	0.751 ± 0.148 ^b^
	n = 160	Numbers of samples (% frequency distribution)	<0.50	8(25%)	8(25%)	6(19%)	0(0%)	0(0%)	
			0.51–1.00	23(72%)	24(75%)	22(69%)	23(72%)	28(88%)	
			>1.00	1(3%)	0(0%)	4(13%)	9(28%)	4(03%)	
Spring	Average AFM_1_ (µg/L)			0.606 ± 0.148	0.664 ± 0.190	0.688 ± 0.247	0.687 ± 0.287	0.625 ± 0.167	0.654 ± 0.037 ^c^
	n = 160	Numbers of samples (% frequency distribution)	<0.50	7(22%)	7(22%)	10(31%)	6(19%)	8(25%)	
			0.51–1.00	25(78%)	24(75%)	19(59%)	21(66%)	23(72%)	
			>1.00	0(0%)	1(3%)	3(9%)	5(16%)	1(8%)	
Summer	Average AFM_1_ (µg/L)			0.416 ± 0.185	0.506 ± 0.202	0.458 ± 0.180	0.507 ± 0.241	0.391 ± 0.251	0.455 ± 0.052 ^d^
	n = 400	Numbers of samples (% frequency distribution)	<0.50	60(75%)	40(50%)	51(64%)	43(54%)	62(78%)	
			0.51–1.00	19(24%)	38(48%)	29(36%)	32(40%)	16(20%)	
			>1.00	1(1%)	2(3%)	0(0%)	5(6%)	2(3%)	
Overall	n = 960	Average AFM_1_ (µg/L)		0.58 ± 0.22	0.64 ± 0.15	0.66 ± 0.19	0.71 ± 0.21	0.64 ± 0.25	0.640 ± 0.053

Statistical analysis: One-way analysis of variance (ANOVA) was applied; to check for significant differences among means of regions and seasons, the Tukey HSD test was used. The results were considered statistically significant at p < 0.05. Means with superscript letters ^a, b, c^ and ^d^ show significant differences between seasons.

**Table 3:** Calibration data for the coefficient of determination obtained by the linear model.

Analyte	Unit	Concentration Range		Slope		Intercept		Coefficient of Determination R^2^	Standard Deviation of Residuals
		Min	Max	Central Value	Slope = 0 (Y/N)	Central Value	Intercept = 0 (Y/N)		
AFM_1_	µg/L	0.125	2.0	0.93	N	0.04	N	0.996	0.044

**Table 4:** Validation parameters of the ELISA method used for the quantification of AFM_1_.

Spiking Levels (µg/L)	No. of Days x No. of Replicates	Median	Repeatability			Intermediate Reproducibility			LOD (µg/L)	LOQ (µg/L)	Recovery (%)	RSD(Rec) Corrected (%)
			SD(r)	CV(r) (%)	R	SD (iR)	CV (iR) (%)	iR				
0.25	6 × 2	0.22	0.021	9.8	0.058	0.053	24.6	0.146	0.10	0.25	86.0	9.6
0.50	6 × 2	0.39	0.032	9.5	0.102	0.044	11.5	0.123	-	-	77.0	15.4
0.75	6 × 2	0.60	0.043	7.0	0.116	0.032	5.4	0.089	-	-	79.3	13.1

SD, standard deviation; (r), repeatability; CV, coefficient of variation; iR, intermediate reproducibility; RSD (rec), corrected relative standard deviation of the recovery.
